# Comparison of irinotecan and oxaliplatin as the first-line therapies for metastatic colorectal cancer: a meta-analysis

**DOI:** 10.1186/s12885-021-07823-7

**Published:** 2021-02-04

**Authors:** Sadayuki Kawai, Nozomi Takeshima, Yu Hayasaka, Akifumi Notsu, Mutsumi Yamazaki, Takanori Kawabata, Kentaro Yamazaki, Keita Mori, Hirofumi Yasui

**Affiliations:** 1 Division of Gastrointestinal Oncology, Shizuoka Cancer Center, 1007 Shimonagakubo, Nagaizumi, Sunto-gun, Shizuoka, 411-8777 Japan; 2 Department of Medical Oncology, Shizuoka General Hospital, 4-27-1 Kita ando, Aoi-ku, Shizuoka City, 420-8527 Japan; 3 Department of Psychiatry, Kitabayashi Hospital, 7-58 Nakamura-cho, Nakamura-ku, Nagoya, Aichi 453-0053 Japan; 4 Department of Psychiatry, Tsukuba Psychosomatics Clinic, 5-12-4, Kenkyu-gakuen, Tsukuba, Ibaraki, 305-0817 Japan; 5 Clinical Research Center, Shizuoka Cancer Center, 1007 Shimonagakubo, Nagaizumi, Sunto-gun, Shizuoka, 411-8777 Japan; 6 Information Management Office, Shizuoka Cancer Center, 1007 Shimonagakubo, Nagaizumi, Sunto-gun, Shizuoka, 411-8777 Japan

**Keywords:** Meta-analysis, Metastatic colorectal cancer, Chemotherapy

## Abstract

**Background:**

Irinotecan (IRI) and oxaliplatin (Ox) are standard therapeutic agents of the first-line treatments for metastatic colorectal cancer (mCRC). Previous meta-analyses of randomized controlled trials (RCTs) showed that treatment with Ox-based compared with IRI-based regimens was associated with better overall survival (OS). However, these reports did not include trials of molecular targeting agents and did not take methods for the administration of concomitant drugs, such as bolus or continuous infusion of 5-fluorouracil, into account. A systematic literature review was performed to compare the efficacy and toxicity profiles between IRI- and Ox-based regimens as the first-line treatments for mCRC.

**Methods:**

This meta-analysis used data from the Cochrane Central Register of Controlled Trials, PubMed, and SCOPUS. The primary endpoint was OS, and the secondary endpoints were progression-free survival (PFS), objective response rate (ORR), and adverse events (AEs).

**Results:**

Nineteen trials involving 4571 patients were included in the analysis. No statistically significant difference was observed between the two groups in terms of OS, PFS, and ORR. There was no significant heterogeneity. Regarding ≥ grade 3 AEs, IRI-based regimens were associated with a high incidence of leukopenia, febrile neutropenia, and diarrhea. Moreover, there was a high incidence of thrombocytopenia and peripheral sensory neuropathy in patients who received Ox-based regimens. In a subgroup analysis, IRI combined with bevacizumab was correlated with a better PFS (HR = 0.90, 95% CI = 0.82–0.98, *P* = 0.02), but not with OS (pooled HR = 0.91, 95% CI = 0.80–1.03, *P* = 0.15).

**Conclusion:**

Although the safety profiles of IRI- and Ox-based regimens varied, their efficacy did not significantly differ. The combination of anti-VEGF antibody and IRI was associated with better PFS compared with anti-VEGF antibody and Ox. Both regimens could be used as the first-line treatments for mCRC with consideration of the patients’ condition or toxicity profiles.

## Background

Currently, colorectal cancer (CRC) ranks fourth for incidence worldwide, with 550,000 deaths recorded annually [1]. Patients with unresectable metastatic CRC (mCRC) have a poor prognosis, with a median overall survival (OS) of 6–8 months, without any therapy. Although chemotherapy is the standard treatment for these patients, the median OS is only 20–25 months [2].

The combination of cytotoxic and molecular targeting agents is currently used as the first-line treatment for patients with unresectable mCRC. Irinotecan (IRI) or oxaliplatin (Ox) combined with fluoropyrimidines (FPs) is considered as the backbone of cytotoxic agents for the treatment of mCRC [3]. In the meta-analysis conducted by Grothey et al. [4], the use of all three active drugs (FPs, IRI, and Ox) for mCRC was associated with a long OS.

First-line treatment is important for patients with mCRC, because the treatment period is the longest of the overall treatment time and significantly affects survival and quality of life (QOL). With regard to the use of cytotoxic agents, several trials investigated the best combination of regimens. Tournigand et al. [5] and Colucci et al. [6] conducted clinical trials to compare the efficacy between 5-fluorouracil (5-FU), folinic acid, and Ox (FOLFOX) regimen and 5-FU, folinic acid, and IRI (FOLFIRI) regimen. Results showed no significant differences between the two regimens. Moreover, Yamazaki et al. [7] compared these regimens with bevacizumab (BEV), a molecular targeting agent. Results showed that the OS did not significantly differ between patients who received FOLFOX + BEV and FOLFIRI + BEV. Hence, both IRI- and Ox-based regimens could be used as the first-line treatments for mCRC.

In clinical practice, physicians generally select the regimen based on adverse events (AEs) caused by the drugs or the patients’ condition. Recent reports showed that physicians preferred Ox-based regimen than IRI-based regimen. Field et al. [8] conducted a survey on the use of chemotherapy for CRC. Results showed that 92.6% of medical oncologists in Australia select Ox-based regimen as the first-line treatment. They reported physicians commonly select this regimen as it has better efficacy and lower toxicity than IRI-based regimen. In addition, Marschner et al. [9] conducted a prospective cohort study and revealed that Ox-based regimen was used as the first-line treatment in 430 out of 605 patients (71.0%) with mCRC.

Previous meta-analyses [10–12] of the first-line treatments for mCRC revealed that Ox-based regimen was correlated with a better OS. However, these studies included trials comparing continuous 5-FU infusion regimen with bolus 5-FU regimen, which is currently considered an inferior method. Moreover, these data were obtained from reports of regimens without molecular targeting agents. Therefore, they might not be applicable in the current clinical practice. Recently, a population-based observational study conducted by Teng et al. [13] reported that IRI-based regimen might be more effective in improving OS than Ox-based regimen. Therefore, it remains unclear which regimen is associated with a better OS. Thus, a meta-analysis based on the current knowledge was performed to compare the efficacy and toxicity between IRI- and Ox-based regimens as the first-line treatments for mCRC.

## Methods

### Search strategy and selection criteria

We searched PubMed, SCOPUS, and the Central Registry of Controlled Trials of the Cochrane Library (CENTRAL) without language restrictions. The last search update was performed on December 17, 2018. The inclusion criteria were as follows: (1) randomized controlled trials comparing IRI- and Ox-based combination regimens as the first-line treatments for mCRC and (2) those using similar agents (IRI- or Ox-based combination regimens) or agents with comparable efficacy based on the results of previous randomized controlled trials. The efficacy of oral FPs was similar to that of continuous infusion of 5-FU. That is, capecitabine + Ox (CapOx) regimen and S-1 + Ox (SOX) regimen were considered as alternative to FOLFOX regimen [14–16]. Similarly, capecitabine + IRI (CapIRI) and S-1 + IRI (IRIS) regimens were considered as alternative to FOLFIRI regimen [17, 18]. (3) Abstracts or unpublished data were included if they had sufficient information on study design, characteristics of participants, interventions, and outcomes.

The search terms used were as follows: (1) terms suggestive of “colorectal” (i.e., “colorect*,” “colon,” “colonic,” “bowel*,” “recta*,” or “rectum”), (2) “cancer” (i.e., “cancer,” “carcinoma*,” “neoplas*,” or “tumor”), (3) “irinotecan” (i.e., “irinotecan,” “camptotecin-11,” or “topotecin”), (4) “oxaliplatin” (i.e., “L-OHP,” “eloxatine,” or “oxaliplatin”), and (5) “randomized trials” (i.e., “ramdomized controlled,”,“randomised,” or “randomly”).

### Data extraction

At least two of three review authors (SK, NT, and YH) independently scanned titles and abstracts to exclude studies that do not meet the criteria. The full text reports were then reviewed for further assessment. Disagreements were resolved via a consensus. Detailed data from eligible trials, such as year of publication, place where the study was conducted, study design, regimens, participants’ information, methodological evaluation, outcomes, and AEs, were extracted. If necessary, the authors were contacted for clarifications.

### Assessment of risk of Bias

At least two of three authors (SK, NT, and YH) independently assessed the risk of bias in the included studies in accordance with the Cochrane Handbook for Systematic Reviews of Interventions [19]. The following domains were assessed: (1) sequence generation; (2) allocation concealment; (3) blinding of participants, personnel, and outcome assessors; (4) incomplete outcome data; (5) selective outcome reporting; and (6) other potential threats to validity. When inadequate details of methodological characteristics of trials were provided, the authors were contacted to obtain further information.

### Statistical analysis

The primary endpoint was OS. The secondary endpoints were progression-free survival (PFS), objective response rate (ORR), and ≥ grade 3 AEs according to the Common Terminology Criteria for Adverse Events version 4.0. Hazard ratios (HRs) and 95% confidence interval (CI), which were considered as relevant effect measures, were directly or indirectly assessed using the given data. When the Kaplan–Meier curve, but not the HR for PFS or OS, was included, we extracted the value using the Engauge Digitizer 10.8 and the method provided by Tierney et al. [20] ORR were compared using odds ratio (OR). The risk difference was used to compare the risk of AEs. Statistical heterogeneity among the studies was assessed using the chi-square test and was expressed with the *I*^*2*^ index [19]. The pooled effect was calculated with the random-effects model. Subgroup analyses were performed according to the combination of agents, which include regimens with or without molecular targeting agents, infusion of 5-FU or oral FPs, and anti-epidermal growth factor receptor (EGFR) or anti-ca (VEGF) antibodies. Since a previous report showed that CapOx plus anti-EGFR antibody might be less effective than FOLFOX plus anti-EGFR [21], we added the subgroup of anti-EGFR antibodies except CapOx + cetuximab. Statistical analyses were performed using the RevMan 5.3 software, which was provided by The Cochrane Collaboration. The corresponding funnel plots were used to examine the effect of publication bias visually. A two-sided *P*-value < 0.05 was considered statistically significant. This study was not registered in any registry although we followed a protocol designed for it.

## Results

### Search results and characteristics of the included trials

**Fig. 1:**
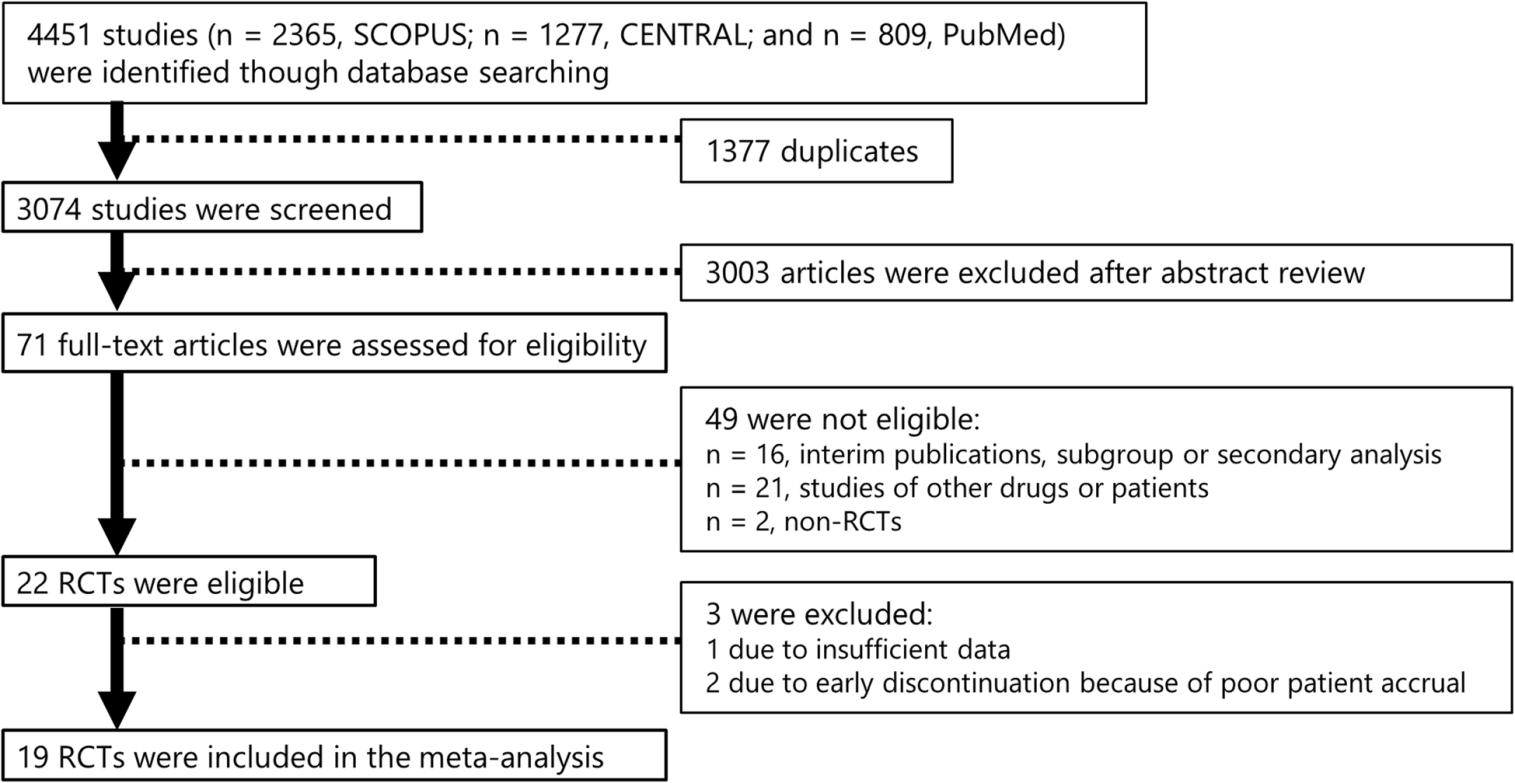
Flowchart of the search process in randomized clinical trials (RCTs)

A total of 4451 potentially relevant studies were retrieved from the initial database search in PubMed, SCOPUS, and CENTRAL. Of these studies, 22 met all the inclusion criteria. However, one trial did not provide sufficient data [22], and two were discontinued early due to poor accrual [23, 24]. Finally, 19 RCTs [5–7, 25–40] with 4571 patients were included in the analysis. A flowchart of the search process is shown in Fig. 1.

Table 1 shows the characteristics of RCTs included in the meta-analysis. Of these, 12 trials (*n* = 3534) used 5-FU infusion regimens; 6 (*n* = 943), oral FPs; and 1, raltitrexed (*n* = 94). Nine RCTs (*n* = 2154) used regimens with molecular targeting agents. Among them, five used BEV and four anti-EGFR antibodies (cetuximab or panitumumab). In total, 2 RCTs were conducted in North America, 13 in Europe, and 4 in Asia. The median age of the participants in all studies was 61–75 years. In one trial [32], only elderly patient aged > 70 years was eligible.

**Table 1:** Characteristics of randomized controlled trials included in the meta-analysis

Study no.	Authors	Region	Year	Trial phase	Primary endpoint	Interventions	Patients	Median OS (months)
1	Grothey et al. [25]	USA	2003	2	AEs	CapOx CapIRI	82 79	NA NA
2	Tournigand et al. [5]	France	2004	3	PFS	FOLFOX FOLFIRI	113 113	20.6 21.5
3	Kalofonos et al. [26]	Greece	2005	2	ORR	Weekly Ox + LV + 5-FU Weekly IRI + LV + 5-FU	148 147	17.4 17.6
4	Comella et al. [27]	Italy	2005	3	ORR	OXAFAFU IRIFAFU	140 133	18.9 15.6
5	Colucci et al. [6]	Italy	2005	3	ORR	FOLFOX FOLFIRI	182 178	15 14
6	Feliu et al. [28]	Spain	2005	2	ORR	Raltitrexed + Ox Raltitrexed + IRI	48 46	NA NA
7	Zheng et al. [29]	China	2006	2	ORR	FOLFOX7 FOLFIRI	30 30	NA NA
8	Bajetta et al. [30]	Italy	2007	2	AEs	TEGAFOX TEGAFIRI	73 68	19 20
9	Seymour et al. [31]	UK	2007	3	OS	OxFU IrFU	357 356	15.4 16.7
10	Rosati et al. [32]	Italy	2010	2	ORR	CapOx CapIRI	47 47	19.3 14
11	Ocvirk et al. [33]	Germany Austria	2010	2	PFS	FOLFOX6 + Cmab FOLFIRI + Cmab	77 74	17.4 18.9
12	Moosmann et al. [34]	Germany	2011	2	ORR	CapOx + Cmab CapIRI + Cmab	92 93	25.5 21.1
13	Schmiegel et al. [35]	Germany	2013	2	PFS	CapOx + BEV CapIRI + BEV	127 128	24.4 25.5
14	Folprecht et al. [36]	Germany Austria	2014	2	ORR	FOLFOX6 + Cmab FOLFIRI + Cmab	56 55	35.8 29
15	Yamazaki et al. [7]	Japan	2016	3	PFS	FOLFOX6 + BEV FOLFIRI + BEV	200 202	30.4 31.4
16	Parikh et al. [37]	USA	2018	2	PFS	FOLFOX6 + BEV FOLFIRI + BEV	188 188	23.9 27.5
17	Carrato et al. [38]	Spain	2017	2	ORR	FOLFOX4 + Pmab FOLFIRI + Pmab	40 40	37 41
18	Yamada et al. [39]	Japan	2018	3	PFS	FOLFOX (CapOx) + BEV S-1 + IRI + BEV	244 243	33.6 34.8
19	Nakayama et al. [40]	Japan	2018	2	ORR	CapOx + BEV CapIRI + BEV	54 53	26.7 28.7

*OS* overall survival, *AEs* adverse events, *PFS* progression-free survival, *ORR* objective response rate, *NA* not available, *CapOx* capecitabine + Ox, *CapIRI* capecitabine + IRI, *FOLFOX and OXAFAFU* 5-FU + folinic acid + Ox, *FOLFIRI and IRIFAFU* 5-FU + folinic acid + IRI, *TEGAFOX* tegafur + folinic acid + Ox, *TEGAFIRI* tegafur + folinic acid + IRI, *BEV* bevacizumab, *Cmab* cetuximab, *Pmab* panitumumab

### Efficacy and toxicity

**Fig. 2:**
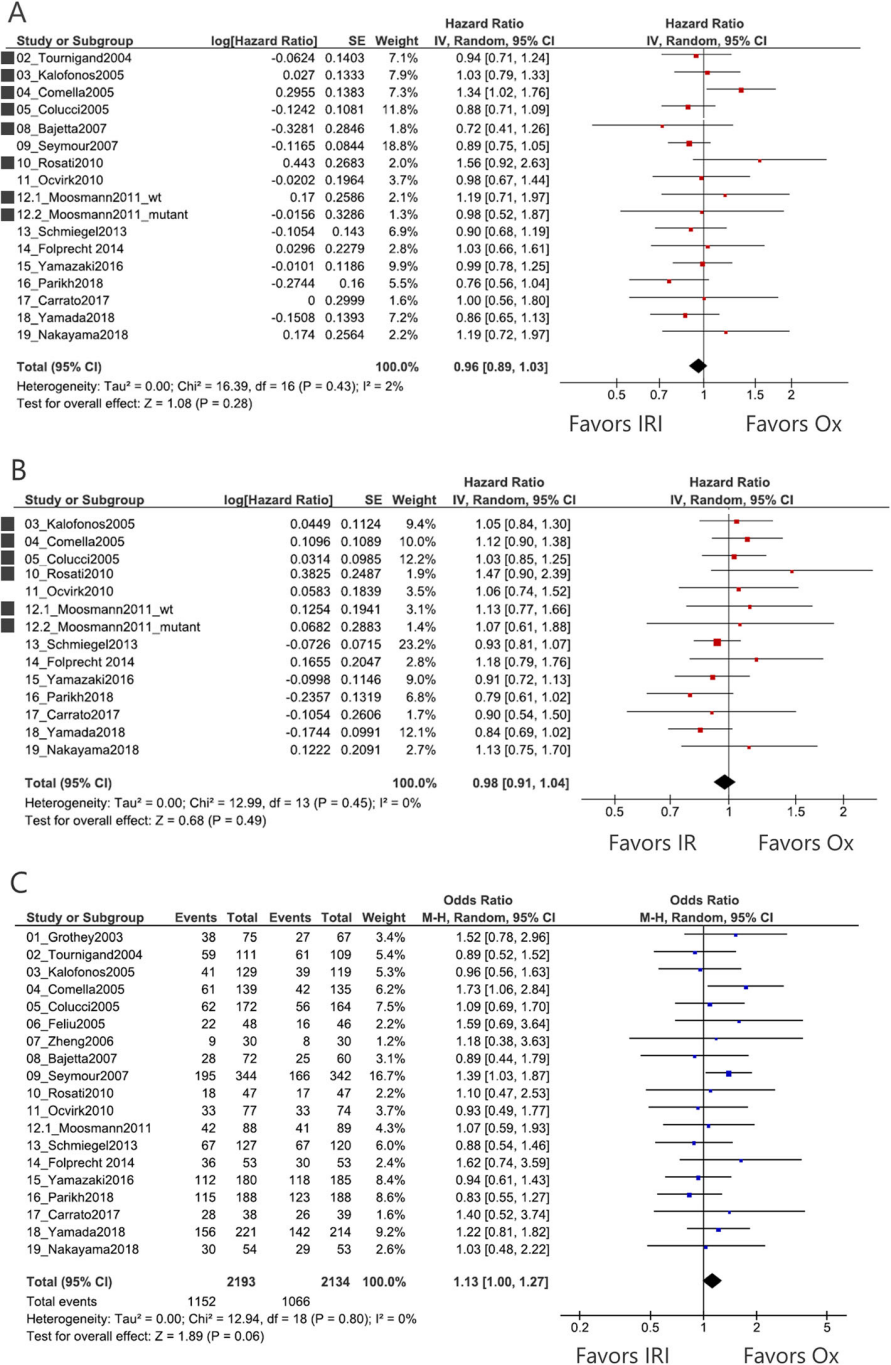
Pooled analyses of each endpoint. Black dot at the left side represents the estimated hazard ratio obtained using the Kaplan–Meier curve. **a** Pooled analysis of overall survival (OS). **b** Pooled analysis of progression-free survival (PFS). **c** Pooled analysis of objective response rate (ORR). IRI, irinotecan; Ox, oxaliplatin; and CI, confidence interval

Sixteen trials included data on OS (Fig. 2a). Since seven RCTs did not describe the HR for OS, we estimated the value using the Kaplan–Meier curve. Results showed no significant difference between IRI- and Ox-based regimens (pooled HR = 0.96, 95% CI = 0.89–1.03, *P* = 0.28), and there was no significant heterogeneity among the trials (*I*^*2*^ = 2%). Thirteen trials included data on PFS (Fig. 2b). We estimated the HR for PFS in six trials using the Kaplan–Meier curve. No significant difference was observed between the two groups (pooled HR = 0.98, 95% CI = 0.94–1.04, *P* = 0.49), and there was no significant heterogeneity (*I*^*2*^ = 0%). All trials included data on ORR (Fig. 2c). There was no significant difference between the two groups. However, Ox-based regimen had a favorable outcome (pooled OR = 1.13, 95% CI = 1.00–1.27, *P* = 0.06). No significant heterogeneity was observed (*I*^*2*^ = 0%).

**Fig. 3:**
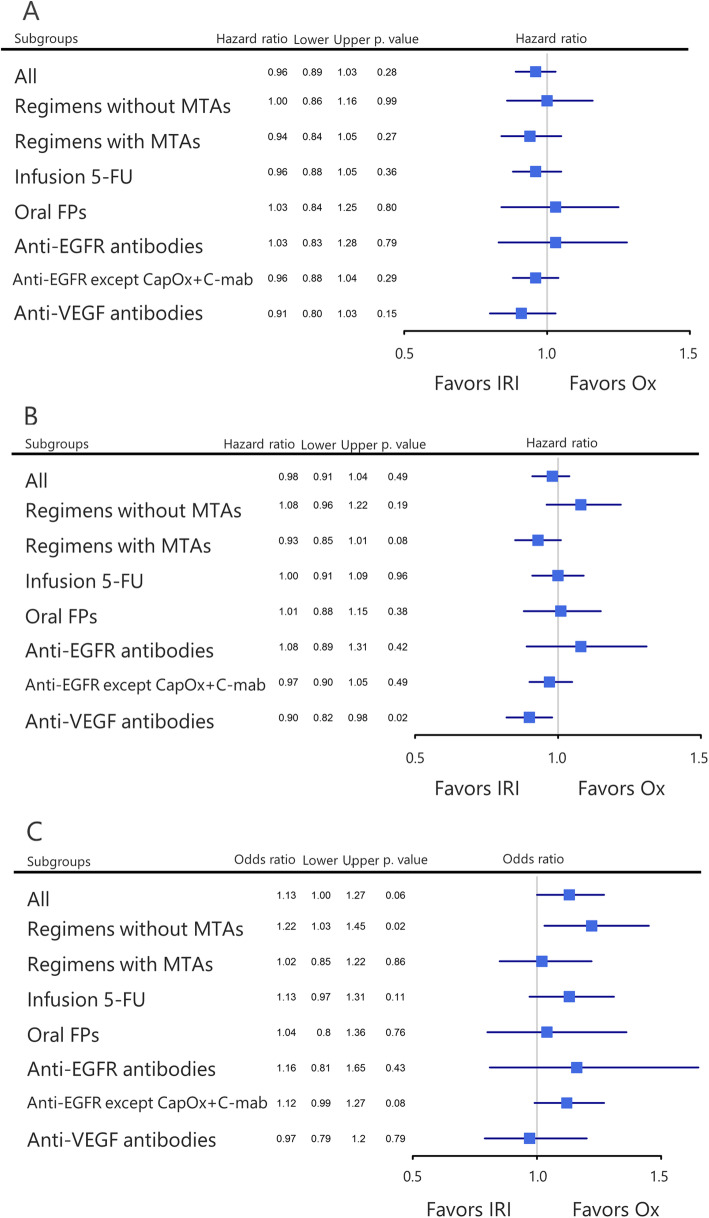
Subgroup analyses according to combination regimens. **a** Pooled analysis of overall survival (OS). **b** Pooled analysis of progression-free survival (PFS). **c** Pooled analysis of objective response rate (ORR). IRI, irinotecan; Ox, oxaliplatin; MTAs, molecular targeting agents; CapOx, capecitabine plus oxaliplatin; C-mab, cetuximab; 5-FU, 5-fluorouracil; FPs, fluoropyrimidines; EGFR, epidermal growth factor receptor; and VEGF, vascular endothelial growth factor

We then performed a subgroup analysis according to the combination regimens. Overall, the OS did not significantly differ between IRI- and Ox-based regimens in all subgroups (Fig. 3a). The combined therapy of IRI and anti-VEGF was associated with a better PFS (pooled HR = 0.90, 95% CI = 0.82–0.98, *P* = 0.02), but not with OS (pooled HR = 0.91, 95% CI = 0.80–1.03, *P* = 0.15) compared with Ox and anti-VEGF combination (Fig. 3b and a). Ox-based regimens, especially those without molecular targeting agents, were associated with a better ORR (pooled OR = 1.22, 95% CI = 1.03–1.45, *P* = 0.02) (Fig. 3c).

**Fig. 4:**
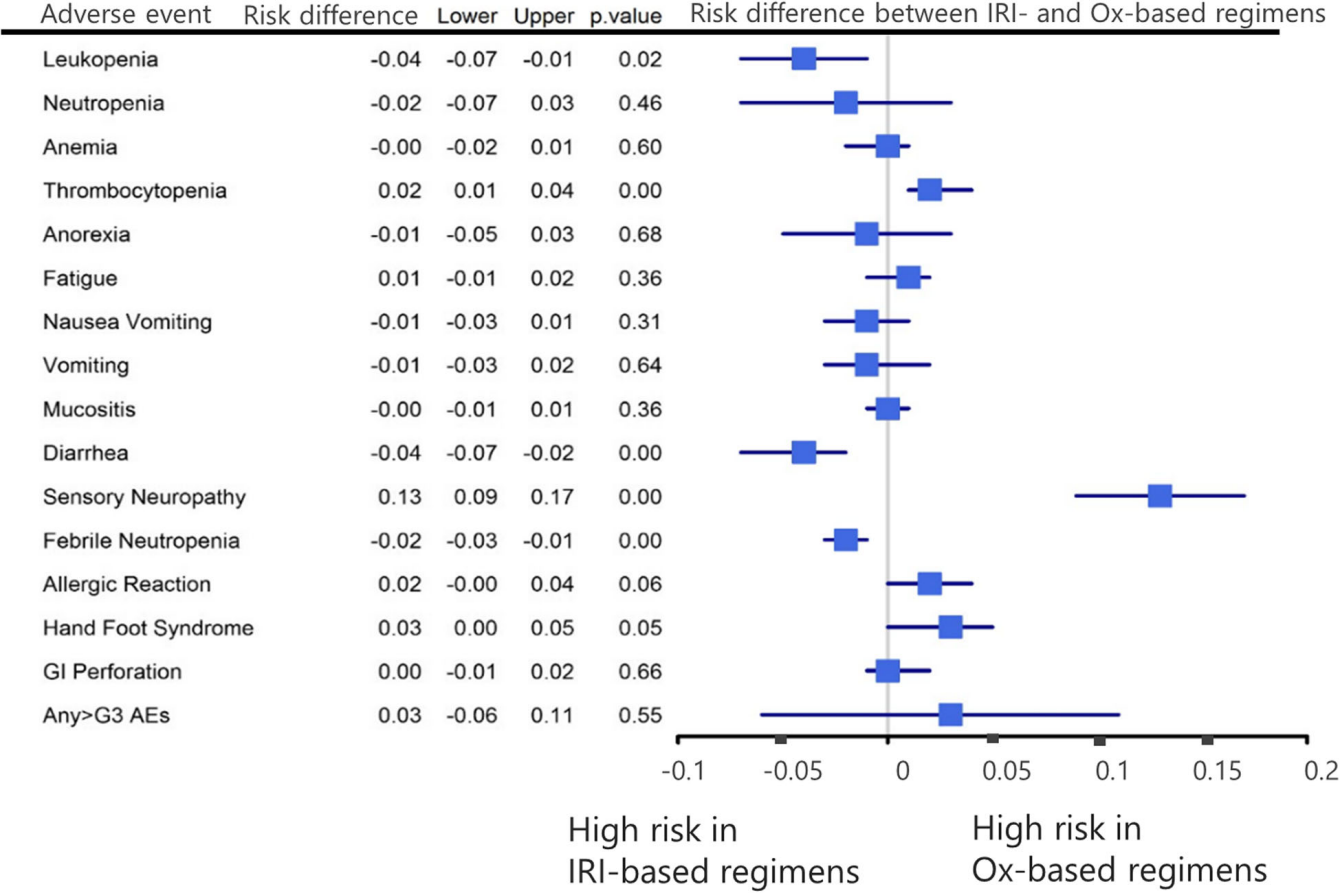
Risk difference in the incidence of ≥ grade 3 AEs between patients who received IRI- and Ox-based regimens. IRI, irinotecan; Ox, oxaliplatin

The incidence of any ≥ grade 3 AEs did not significantly differ between patients who received the two regimens. Thrombocytopenia, peripheral sensory neuropathy, hand–foot syndrome, and allergic reaction were significantly correlated with Ox-based regimens. Meanwhile, leukopenia, diarrhea, and febrile neutropenia were commonly observed in patients who received IRI-based regimens (Fig. 4).

### Assessment of risk of Bias

**Fig. 5:**
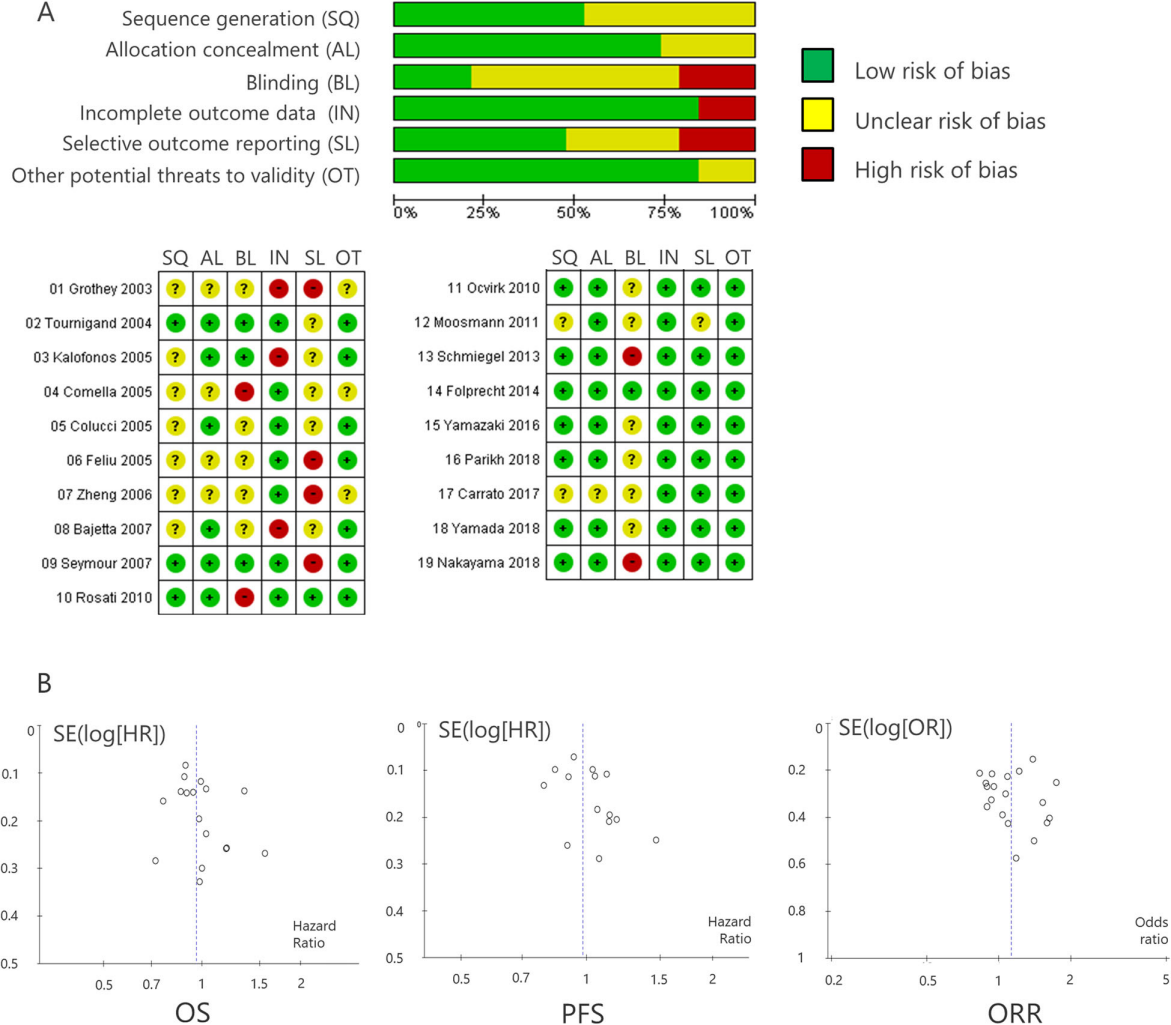
Assessment of risk of bias. **a** Risk of bias assessment for each included RCT. The bar chart indicates the distribution of risk-of-bias judgments across the domains. **b** The funnel plot of each effect size in terms of overall survival (OS), progression-free survival (PFS), and objective response rate (ORR). SQ, sequence generation; AL, allocation concealment; BL, blinding; IN, incomplete outcome data; SL, selective outcome reporting; OT, other potential threats to validity; SE, standard error; HR, hazard ratio; OR, odds ratio

Risk of bias assessment is shown in Fig. 5. Overall, the quality of evidence in this study was moderate according to the risk of bias assessment (Fig. 5a). The funnel plot of each effect size in terms of OS, PFS, and ORR was symmetrical, with a similar number of studies on either side of the summary treatment effect. This result indicated a lack of major publication bias (Fig. 5b).

## Discussion

Based on the current expert panels of guidelines [3, 41, 42], there is no preferred regimen that can be used as the initial therapy for mCRC. Hence, IRI- or Ox-based regimen can be an alternative. In clinical practice, the regimen is selected based on efficacy of the regimens, physicians’ experience, and profiles of AEs.

Our analysis showed that there was no significant difference in terms of OS between patients who received IRI- and Ox-based regimens as the first-line treatments for mCRC. This is the first meta-analysis that included treatments containing molecular targeting agents and clinically comparable combination of regimens. Therefore, the results of this study may be more applicable to current clinical practice than those of previous studies. The absence of significant heterogeneity between trials in terms of OS, PFS, and ORR indicated consistent results. Previous reports [10–12] showed that Ox-based regimen was more preferred in terms of OS. This discrepancy in results may be attributed to the selection criteria of the trials. That is, previous studies included trials comparing FOLFOX with IFL (bolus 5-FU + IRI) regimen [43, 44]. However, Delaunoit et al. [45] showed that bolus 5-FU + IRI or Ox could be associated with severe gastrointestinal toxicity and high mortality rates. Thus, excluding these trials was appropriate for the evaluation of efficacy and toxicity between the two regimens.

In the subgroup analysis of regimens without molecular targeting agents, Ox-based regimen was associated with a better ORR. However, the PFS and OS did not significantly differ. In colorectal cancer, the correlation between ORR and OS is not clearly elucidated [46]. Hence, our results were not conflicting. In addition, in this subgroup, six of ten trials [6, 26–29, 32] used ORR as the primary endpoint, and four [6, 27, 29, 32] studies hypothesized that Ox-based regimen is associated with a better ORR. Thus, the evaluation of ORR might be biased in these trials.

In the subgroup analysis of regimens with molecular targeting agents, the combination of anti-VEGF antibody and IRI was more likely to be associated with better PFS and OS. Ren et al. [47] reported same results in their meta-analysis including RCT and observational studies. Previous studies [48, 49] suggested that SN38, an active metabolite of IRI, had multiple anti-angiogenic effect through inhibition of VEGF production via inhibition of HIF-1α and a direct effect on endothelial cells. Thus, anti-VEGF antibody and IRI might work synergistically. As a growing number of active compounds have become available as second- or subsequent line chemotherapy for colorectal cancer in recent years, we speculated that prolonged PFS might not directly lead to significantly longer OS.

With regard to the results of AEs, though details of each AE was different from each other, the incidence of any ≥ grade 3 AEs did not significantly differ between patients who received the two regimens. Therefore, it may be reasonable for physicians to select each regimen according to AE profiles. However, previous systematic reviews including any grade of AE [12, 47] reported that the number of incidence of nausea/vomiting and mucositis are more observed in IRI-based regimens without molecular targeting agents than Ox, and bleeding event and venous thromboembolism are more observed in IRI + anti-VEGF regimens compared with Ox + anti-VEGF. Thus combination agents may affect the toxicity profile. Yamazaki et al. [7] revealed that the FACT-C OQL score was more likely worse in patients treated with FOLFOX + BEV than in those treated with FOLFIRI + BEV. Moreover, a previous report indicated that peripheral sensory neuropathy lasts for a long period and affects QOL after treatment [50]. Thus, physicians should consider QOL during not only the first-line treatment but also the overall survival time when selecting the regimens.

This study had some limitations. That is, it is a meta-analysis. Moreover, there were no data on race, sex, primary site of the tumor, and status of *RAS* and *BRAF* genes. Thus, our data must be applied with caution to individual patients in clinical practice. Recently, some reports showed that the molecular subtypes or chromosomal variation of CRC might affect the efficacy of IRI- and Ox-based regimens [51, 52]. Thus, in the future, suitable cytotoxic agents should be selected according to biomarker profile.

## Conclusions

The efficacy of IRI- and Ox-based regimens when used as the first-line treatments for mCRC did not significantly differ. In subgroup analysis, the combination of anti-VEGF antibody and IRI was associated with better PFS. Both regimens could be used in clinical practice with consideration of the patients’ condition or toxicity profiles.

## Data Availability

The datasets used and/or analyzed during the current study are available from the corresponding author on reasonable request.

## Abbreviations

IRI: Irinotecan
Ox: Oxaliplatin
mCRC: Metastatic colorectal cancer
RCT: Randomized controlled trial
OS: Overall survival
PFS: Progression free survival
ORR: Objective response rate
AEs: Adverse events
HR: Hazard ratio
FPs: Fluoropyrimidines
QOL: Quality of life
CENTRAL: The Central Registry of Controlled Trials of the Cochrane Library
CI: Confidence interval
EGFR: Epidermal growth factor receptor
VEGF: Vascular endothelial growth factor
CapOx: Capecitabine + oxaliplatin
CapIRI: Capecitabine + irinotecan
IFL: Bolus 5-FU + irinotecan
5-FU: 5-fluorouracil
BEV: Bevacizumab
IRIS: S-1 + irinotecan, FOLFOX and OXAFAFU, 5-FU + folinic acid + oxaliplatin
FOLFIRI and IRIFAFU: 5-FU + folinic acid + irinotecan
TEGAFOX: Tegafur + folinic acid + oxaliplatin
TEGAFIRI: Tegafur + folinic acid + irinotecan
NA: Not available
MTAs: Molecular targeting agents
